# Pioneer factors in viral infection

**DOI:** 10.3389/fimmu.2023.1286617

**Published:** 2023-10-09

**Authors:** Eva Neugebauer, Aura M. Bastidas-Quintero, Daniel Weidl, Florian Full

**Affiliations:** ^1^ Institute of Virology, University Medical Center, Faculty of Medicine, University of Freiburg, Freiburg, Germany; ^2^ Spemann Graduate School of Biology and Medicine (SGBM), University of Freiburg, Freiburg, Germany; ^3^ Faculty of Biology, University of Freiburg, Freiburg, Germany; ^4^ German Consulting Laboratory for Herpes-Simplex Virus (HSV) and Varizellla-Zoster Virus (VZV), Medical Center, University of Freiburg, Freiburg, Germany; ^5^ Institute for Clinical and Molecular Virology, University Hospital Erlangen, Erlangen, Germany

**Keywords:** pioneer factor, virus, DNA-virus, herpesvirus, development, transcription factor, DUX4

## Abstract

Pioneer factors are transcription factors sharing the fascinating ability to bind to compact chromatin and thereby alter its transcriptional fate. Most pioneer factors are known for their importance during embryonic development, for instance, in inducing zygotic genome activation or cell fate decision. Some pioneer factors are actively induced or downregulated by viral infection. With this, viruses are capable to modulate different signaling pathways resulting for example in MHC-receptor up/downregulation which contributes to viral immune evasion. In this article, we review the current state of research on how different viruses (Herpesviruses, Papillomaviruses and Hepatitis B virus) use pioneer factors for their viral replication and persistence in the host, as well as for the development of viral cancer.

## 1 Introduction

Pioneer transcription factors are a rather heterogeneous group of transcription factors that share the unique ability to bind to and change the transcriptional fate of compacted chromatin. In eukaryotic cells, most of the genomic DNA is compacted in nucleosomes to save space and prevent unneeded genes from expression. Whereas “classical” transcription factors are only capable to bind to non-compacted DNA, pioneer factors bind in a sequence specific manner to short DNA stretches exposed at the surface of nucleosomes (1). By doing so, pioneer factors can reverse the fate of compacted DNA by attracting other transcription factors, histone modifying enzymes, DNA-methyltransferases and transcriptional cofactors leading to opening of chromatin (2). Thereby, pioneer factors can have activating, as well as repressing effects on transcription. Whereas pioneer factors pave the way for the opening of chromatin by recruitment of other factors, they are usually not needed for the transcriptional process itself.

Most pioneer transcription factors play important roles in embryonic development (3). In mammalian oocytes for example, transcription is generally halted and almost all genes are silenced. However, after fertilization the newly formed zygote has to go through a process called maternal to zygotic transition (MZT) to activate the diploid genome and enable transcription from the genome. During MZT, maternal mRNAs and proteins stored in the oocyte are gradually degraded and reprogramming of the zygote into a totipotent state starts with the so called zygotic genome activation (ZGA). Several pioneer factors have been shown to be essential for the initiation of transcription during ZGA. In mammals for example the pioneer factors mouse double homeobox (Dux)/human double homeobox 4 (DUX4), NANOG, human nuclear transcription factor Y (NFY) and human Octamer-binding transcription factor 4 (OCT4) have been demonstrated to be critical for ZGA (4–6). Experimental abrogation of one of the factors results in abnormalities in further development of the embryo. Other examples for the potency of pioneer factors are the roles of PU.1 in deciding the cell fate in the hematopoietic lineage or Neurod1 and Ascl1 (Mash1) in neural development (7).

Numerous reviews in the past years focused on the role of pioneer transcription factors during embryonic development and in cancer (1, 3, 7–9). In addition, there are several reviews summarizing the detailed molecular mechanisms of how pioneer transcription factors are able to bind to nucleosome compacted DNA (1–3). In this review we want to focus on the role of pioneer transcription factors during viral infection, a topic that has not been addressed to date. Whereas some pioneer factors are actively induced by viruses, others are potently downregulated upon infection. We hypothesize and provide evidence that viruses use pioneer factors to alter transcription from the viral genome and from the host genome to promote viral replication and oncogenesis. This exploitation of pioneer factor function might have implications for both lytic viral replication/reactivation as well as for the development of virus-associated cancer.

Viruses are important pathogens that, by definition, rely on host cells for their replication and propagation. Upon infection of the host cell, viruses have evolved numerous mechanisms to promote viral replication. First, viruses need to prevent activation of the host innate and adaptive immunity. Second, the virus has to establish specialized replication compartments that provide an environment allowing replication of the viral genome. Viral replication compartments are either located inside the nucleus as it is the case for most DNA-viruses and some RNA viruses like Influenza viruses or Bornaviruses. An alternative strategy are viral replication compartments in the cytoplasm. Examples for cytoplasmic replication are most RNA-viruses and some DNA-viruses like Poxviruses. Most DNA-viruses replicate in the nucleus since this is the place where cellular DNA replication takes place. Therefore, the nucleus provides most of the infrastructure needed for viral DNA replication like dNTPs, cellular enzymes for nucleotide metabolism as well as enzymes for transcription. One notable exception are large nucleocytoplasmic DNA-viruses like Poxviruses or Asfarviruses that replicate in the cytoplasm. This comes with the cost of a big genome size, since the virus has to encode for most genes that are needed for genome replication. In addition, it has been shown that poxviruses also recruit cellular transcription factors like YY1, SP1, and TATA binding protein into cytoplasmic replication compartments, although the consequences for viral replication have not been assessed (10).

DNA-viruses that replicate in the nucleus include families with important pathogens like Adenoviruses, Herpesviruses, Papillomaviruses and Polyomaviruses. For DNA-viruses, it is important to discriminate between lytic viral replication that leads to production and spread of viruses, and persistent infection that is characterized by limited viral gene expression and that can ultimately result in transformation of infected cells. Members of the Herpesvirus, Papillomavirus and Polyomavirus families are associated with cancer in humans as the viral infection results in transformation of cells with uncontrolled cell growth (11–13). Viral cancer is a consequence of longtime viral persistence and expression of viral oncoproteins. Herpesviruses are special regarding persistence compared to other DNA-viruses by establishing lifelong latency in the infected host. Upon infection, herpesviruses are disseminated by lytic replication before establishing a life-long latency in long-lived cells like neurons or memory B-cells. After years of latency with minimal viral gene expression, the viruses can reactivate in response to a number of external and intrinsic stimuli and cause disease (14).

In this paper, we will focus on Herpesviruses, Papillomaviruses and Hepatitis B virus and how they use pioneer factors to modulate replication, tumorigenesis and latency. Importantly, pioneer factors also contribute to viral immune evasion, for example, by up- or down-regulation of cell surface receptors such as MHC-receptors.

### 1.1 Pioneer transcription factors

**Figure 1:**
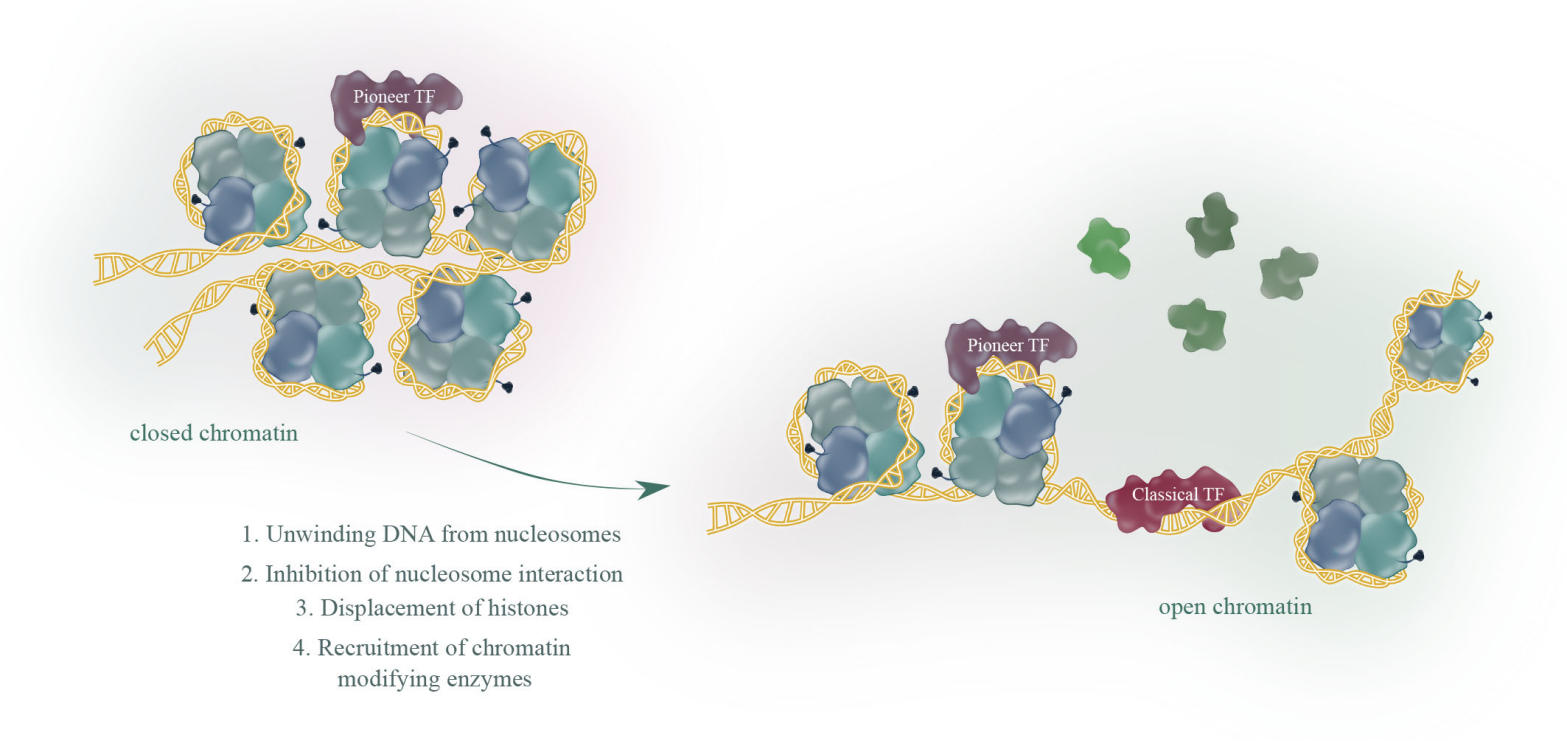
Pioneer transcription factors (TF), in contrast to classical TF, share the ability to bind to closed chromatin. The current model proposes binding of a pioneer factor, which opens chromatin through various mechanisms to allow classical TF binding and subsequent transcription.

There are about 2000 transcription factors encoded in the human genome (15). Most eukaryotic transcription factors can only bind to open chromatin and are not able to initiate transcription from heterochromatin. However, in some situations, for example during development or cell differentiation, a large proportion of the transcriptional fate needs to be changed. This is where a subset of transcription factors, the so called pioneer factors, come into play. Pioneer factors have the intriguing ability to bind to nucleosomes and are therefore able to bind to heterochromatin (closed chromatin). There are several excellent reviews published (1–3, 16) focusing on how pioneer factors are able to open chromatin and alter transcription. An overview of pioneer factor families including functions is summarized in **Table 1**. Despite a lot of progress in the past years in elucidating the exact mechanism of how individual pioneer factors work, there are still aspects that are not fully understood. Although pioneer transcription factors are highly diverse in their structure, all pioneer factors are able to bind to DNA/nucleosomes. DNA-binding domains of pioneer factors comprise a number of different domains like winged-helix domains (e.g. Fox-family factors), homeobox domains (e.g. DUX4) or a high-mobility group (HMG-) domain (Sox-family factors). The structural aspects of pioneer transcription factors were recently summarized in an excellent review by Kagawa and Kurumizaka (17). The exact binding site on the nucleosome differs between the individual pioneer factors, being at the edge or around the nucleosome, close to or directly to the dyad axis. Interestingly, some pioneer factors, such as Cbf1 in *S.cerevisiae* are reported not just bind the DNA, but also with one domain to histones, which stabilizes the pioneer factor binding (18, 19). After binding to the nucleosome, several mechanisms have been reported for the establishment of open chromatin by pioneer factors as depicted in **Figure 1**. These mechanisms range from partial unwinding DNA from nucleosomes, inhibition of nucleosome interactions, displacement of histones to recruitment of chromatin modifying enzymes and are diverse for pioneer factors of different families. In addition, there are further factors that contribute to the transcriptional fate of chromatin in the presence of pioneer factors. For example, there are certain histone modifications that can act as a barrier or promote pioneer factor binding, for example, H3K9me3 has been shown to hamper the binding of OCT4, SOX2, Klf4 and c-Myc (20). This is also reflected by the fact that it is still the cellular context that determines which binding sites the pioneer factor will actually bind to *in vivo*. For example, it has been shown that cell-type specific chromatin structure determines the binding of the pioneer factor PU.1 (21) and Zelda (22). Overall, the detailed mechanisms are yet not fully understood and the current knowledge is summarized in the excellent reviews by Zaret, Larson et al. and Balsadore et al. (1–3).

**Table 1:** Pioneer factor family, DNA binding domain and function of pioneer factors modulated during viral infection.

Pioneer factor family	DNA binding domain	Pioneer factor members	Function
Fox	Winged-helix	FoxA, FoxO	Development, cell and tissue differentiation and homeostasis, cell cycle control, metabolism, stress response
ETS	Winged helix-turn-helix	PU.1, ELF1-4	Immunity, development, cell differentiation and homeostasis and cancer
SOX	High-mobility-group	SOX2,9	Induction, Maintainance pluripotency
POU	POU binding domain	OCT4	Induction Pluripotency
KLF	Zinc finger	KLF4, KLF13, KLF15	Proliferation, Differentiation, Apoptosis, Tissue development and homeostasis
GATA	Two zinc finger motif	GATA1-6	Cell differentiation
PAX	Paired domain	PAX1-9	Tissue and organ development
RUNX	Runt	RUNX1-3	Embryonic development
DUX4	homeoboxdomain	DUX4	Embryonic development

In this review, we focus on pioneer transcription factors that have been shown to be regulated and play a role in viral infections. The pioneer transcription factors we took a look at in this review in the context of viral infection are listed in the following table, with their DNA binding domain and function.

### 1.2 Herpesviruses and pioneer factors

**Figure 2:**
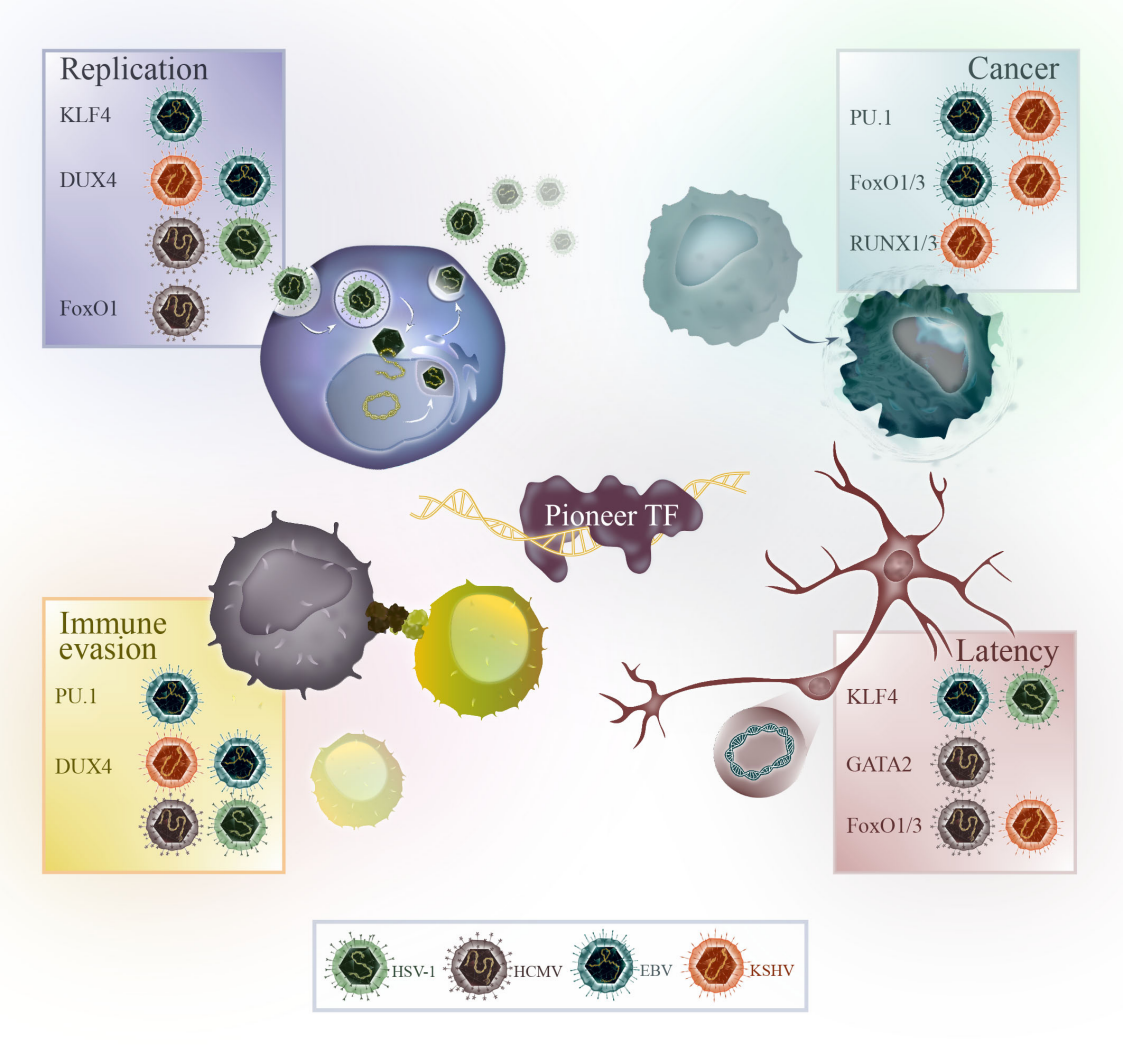
Overview of the pioneer factors modulated by HSV-1 (green), HCMV (brown), EBV (blue) or KSHV (orange) for viral replication, cancer progression, immune evasion or in the context of latency.

Herpesviruses are a family of dsDNA-viruses characterized by their ability to establish lifelong infections in their hosts. Human herpesviruses can cause different diseases, including oral and genital herpes, chickenpox, shingles and infectious mononucleosis. Herpes simplex virus-1 (HSV-1), herpes simplex virus-2 (HSV-2) and varicella zoster virus (VZV) are alphaherpesviruses, human cytomegalovirus (HCMV) and roseoloviruses (HHV-6A/B, HHV-7) are betaherpesviruses, and Epstein-Barr virus (EBV) and Kaposi’s sarcoma-associated herpesvirus (KSHV) are gammaherpesviruses. Herpesviruses have a unique life cycle that includes both lytic and latent phases. During the lytic phase, the virus actively replicates and causes symptoms in the host. After the initial infection, herpesviruses are not cleared and enter latency in the host. During latency, the virus normally does not cause symptoms and can remain dormant for long periods of time. The gamma herpesviruses EBV and KSHV are special in their ability to cause cancer in humans. A summary of the pioneer factors, which are modulated by herpesviruses for their replication, tumorigenesis, latency and immune evasion are illustrated in **Figure 2**.

Infection with EBV usually occurs in early childhood and is asymptomatic, but if infection occurs later in life, the virus may cause infectious mononucleosis. EBV establishes latency in long-lived memory B-cells which results in lifelong persistence in infected individuals. EBV is associated with a number of human cancers like Burkitt-lymphoma, nasopharyngeal carcinoma, gastric cancers and Hodgkins lymphoma (23). EBV has been shown to modulate many different pioneer factors, namely FoxO1/3, KLF4, PU.1, RUNX (24) and SOX9 (25). The expression pattern of latency-associated viral genes may vary. In general, latency is divided into type I latency, which is found in Burkitt’s lymphoma and gastric cancer, type II latency in Hodgkin’s lymphoma and nasopharyngeal carcinoma, and type III latency in lymphoblastoid cell lines (26) EBNA2, which is expressed during type III latency, transactivates late membrane protein 1 (LMP1). This activation is mediated by interaction with the cofactors recombination signal binding protein Jκ (RBPJk) and pioneer factor PU.1 to direct EBNA2 to the LMP1 promotor (27, 28). LMP1 is known as the major oncogene of EBV and has been shown to play an important role in B-cell transformation. In addition, EBV’s non-coding RNA EBER2 has been shown to associate with UCHL1 mRNA, which in a complex induces the pioneer PU.1. This is thought to help transform resting B cells into lymphomas (29). PU.1 has also been implicated in the downregulation of MHCII after EBV infection by LMP2A (30). Furthermore, the two FoxO proteins FoxO1 and FoxO3 are downregulated by LMP1 and LMP2A as well as by the PI3K/Akt and MAPK/ERK pathway. This regulation of FoxO protein expression promotes tumorigenesis by promoting cell transformation, suppressing DNA repair and inhibiting apoptosis (31–36), indicating that EBV needs to downregulate FoxO1/3. Other pioneer factors that influence B cell growth and are modulated by EBV are RUNX1 and RUNX3. Gunnell et al. showed that the latency proteins EBNA2 and EBNA3B/C specifically alter RUNX1/3 expression to influence B cell growth. EBNA2, 3B/C activate RUNX3 by binding to an upstream super-enhancer. Activation of RUNX3 leads to a feed-forward loop in which RUNX1 expression is repressed to manipulate B-cell growth. In addition to the repression by RUNX3, RUNX1 expression is further regulated though inhibition by EBNA3B/C and activation by EBNA2 (24). Furthermore, EBV is regulating the pioneer factor KLF4. EBV has been shown to increase KLF4 levels through the immediate early protein BZLF1. Mechanistically, BZLF1 inhibits the methyltransferase METTL3, which leads to a decrease of the mRNA modification m^6^A of KLF4. This provides enhanced stability of the KLF4 mRNA for enhanced KLF4 levels, which promotes lytic replication (37). Along this line, KLF4 has also been suggested to contribute to EBV reactivation during epithelial differentiation, when KLF4 is expressed together with BLIMP1. Van Sciver et al. have been shown that KLF4 and BLIMP1 activate the promotors of the genes encoding for the IE proteins BZLF1 and BRLF1, which results in lytic reactivation of the virus (38).

Primary HSV-1 infection usually occurs early in life, mainly in the oral mucosa. After entry into the cell, the viral genome is released into the nucleus where HSV-1 transcription and replication take place. Transcription of HSV-1 genes occurs in a cascade of immediate-early, early and late genes. After initial infection, the virus establishes latency in neurons. During latency, HSV-1 is maintained in an episomal form and only latency-associated transcripts (LATs) are expressed. When HSV-1 is reactivated, it switched back to lytic infection. The entire mechanism of reactivation is not fully understood, but one of the stimuli associated with HSV-1 reactivation is cellular stress. The glucocorticoid receptor and stress-induced transcription factors, including the pioneer factor KLF4, have been shown to play a role in the activation of infected cell protein 4 and 0 (ICP4 and ICP0) (39, 40). Glucocoticoid receptor treatment results in KLF4 expression which binds to the ICP4 promoter and activates its transcription. ICP4 is an immediate-early protein of HSV-1 which transactivates viral genes and is needed for productive lytic replication. A similar mechanism was also reported to play a role in the reactivation of bovine herpesvirus 1 (BoHV-1), an alphaherpesvirus of cows, indicating that this is conserved across herpesviruses of different species (41). Another pioneer factor involved in HSV-1 infection is RUNX1, which has been reported to play a role in inhibiting the transcription of several viral genes, thereby limiting infection (42). RUNX1 is a transcription factor involved in the differentiation of sensory neurons. The HSV-1 genome is enriched for binding-sites of RUNX1 and binding of overexpressed RUNX1 prevents HSV-1 gene expression and lytic replication in an *in vitro* model of HSV-1 in neuroblastoma cells (42). This indicates, that RUNX1 contributes to the establishment of latency in neuronal cells. Taken together, both KLF4 and RUNX1 are involved in regulating gene expression from the EBV and HSV-1 genome, which contributes to different aspects of latent and lytic replication. Whereas the regulation of latency is more complicated for EBV with different transcriptional programs in different types of latency, the data hint at an involvement of the pioneer factors KLF4 and RUNX1 in the switch between latent and lytic infection of HSV-1.

HCMV is a beta-herpesvirus that causes a variety of diseases in immunocompromised individuals. In addition, HCMV is a major cause of birth defects upon congenital infection of the mother. Like other herpesviruses, HCMV has two phases, lytic and latent infection. To maintain latency, the pioneer factor GATA2 (43) has been described to play an important role in the expression of the latency-associated gene LUNA and, in some HCMV isolates, UL144. LUNA is a deSUMOylase that is involved in dispersion of PML nuclear bodies and HCMV viruses with LUNA mutations in the deSUMOylation motif show reduced levels of reactivation of latent HCMV. In addition, for HCMV reactivation, the pioneer factors FoxO1 and 3a have been described as critical for the activation of immediate early genes by binding and activating the alternative immediate early promotor (44). In addition, FoxO1 has been described to play an important role in viral transcription and replication (45, 46). Hale et al. showed that the major immediate –early promoter of HCMV that is silenced in latenly infected hematopietic stem cells can be activated by alternative FoxO binding sites. The activation leads to initiation of the lytic cascade and reactivation of the virus (44). Another pioneer factor modulated by HCMV is SOX2. Wu et al. reported that IE1 is downregulating SOX2 in neural progenitor cells, which might be a mechanism of HCMV damaging the developing nervous system (47). Recently it was also shown that in glioma the HCMV gene expression is modulated by SOX2 and subsequent PML downregulation (48).

KSHV is an oncovirus that can cause a rare cancer called Kaposi’s sarcoma (KS), primary effusion lymphoma (PEL) and multicentric Castleman’s disease (MCD) in immunocompromised patients. In PEL cells, the pioneer factor PU.1 is downregulated. This downregulation has been shown to promote cell growth and inhibit apoptosis, which promotes PEL development (49). Another pioneer factor that is important for KSHV is FoxO1. But in contrast to HCMV where FoxO1 promotes reactivation, it has been reported that FoxO1 is important for maintaining latency of KSHV. Inhibition of FoxO1 increases levels of reactive oxygen species that lead to KSHV reactivation, and KSHV can directly upregulate FoxO1 through vFLIP and miRNAs to induce cell proliferation (50, 51).

One pioneer factor is quite special for herpesviruses, as all subfamilies show conserved induction of this protein. This pioneer factor, called DUX4, is known for its role during embryonic development, where it induces zygotic genome activation. In healthy individuals, DUX4 is exclusively expressed in the 2-8 cell stage of early embryonic development. Zygotic genome activation is a crucial event in the maternal to zygotic transition, the switch between limited gene expression in the resting oocyte and genome wide activation of transcription in the zygote. After that short period of expression, the DUX4 locus is epigenetically silenced. However, rarely DUX4 expression gets activated beyond the 8-cell stage and then can cause disease. DUX4 expression in skeletal muscle cells is the cause of facio-scapulo-humeral muscle dystrophy (FSHD) and aberrant expression of DUX4 after the 2-8 cell stage in development causes Bosma-Arhinia Microphtalia Syndrome. In addition there are reports of DUX4 expression in human cancer. Interestingly, we and other could show, that DUX4 is induced by herpesviral infection (52–54). DUX4 is expressed upon lytic replication of herpesviruses by viral proteins, and knockout of DUX4 blocks replication of HSV-1 and HSV-2 almost completely. It is possible that the herpesviruses induce DUX4 to prevent silencing of the viral genome and promote viral replication, basically mimic an embryonic ZGA-like state in the cell.

### 1.3 Papillomaviruses and pioneer factors

Human papillomaviruses (HPV) are non-enveloped dsDNA-viruses that include over 200 different genotypes. High-risk HPV genotypes can cause a variety of cancers, including anal, penile, and head and neck cancers, but the most common type is cervical cancer. The replication cycle of HPV is linked to the differentiation of epithelial cells. HPV infects dividing basal epithelial cells. As the viral genome is passed on to the daughter cells it is carried along to the upper layer of epithelial cells. This allows a regulated progression in HPV’s replication cycle, in which infection and persistence of the viral DNA takes place in basal layer cells, in the middle layer of spinous layer cells viral genome amplification takes place and in the upper granular cells virions are assembled. HPV has an episomal genome that can be divided into three distinct regions. One region encodes the early (E) proteins, another region of the HPV genome carries the late (L) genes encoding the two capsid proteins L1 and L2, and the third region is the long control region (LCR), which controls viral replication and transcription.

About 5% of all human cancers are caused by HPV, mainly by HPV16 and HPV18. However, progression to cancer after HPV infection is actually a rare event, as in most cases the virus is cleared. The key players in HPV tumorigenesis are the early proteins E6 and E7. These two proteins act as oncogenes, enabling uncontrolled cell proliferation, evading apoptosis and inducing chromosomal instability. These proteins have also been shown to modulate the pioneer factors KLF4, SOX2 and OCT4 in the context of tumourigenesis.KLF4 is dysregulated in many different types of cancer and can act here via tumor suppressing or oncogenic function (55, 56). However, in HPV-mediated cancers, there is evidence that KLF4 modulation is mediated by the two HPV proteins E6 and E7. Gunasekharan et al. reported that KLF4 levels are increased in HPV+ cells through post-transcriptional modulation of KLF4 by E7 by suppressing the cellular miRNA miR-145 (57), which normally targets HPV E1 and E2. In addition, E6 inhibits KLF4 SUMOylation and phosphorylation (58). They also showed that KLF4 upregulation not only plays an important role in keratinocyte differentiation and cell cycle progression, but also in the regulation of viral transcription by binding to the LCR together with Blimp1 (58). KLF4 has also been reported to play a role in epithelial-mesenchymal transition (EMT).Other pluripotency and pioneer factors upregulated by HPV include OCT4 (59–61) and SOX2 (62). Oct4 levels are generally upregulated in cervical cancer and even more upregulated in HPV+ compared to HPV-negative cancers (59–61). E7 has been shown to upregulate and directly bind to Oct4, modulating its transcriptional pattern. Recently, the mechanism by which E7 upregulates OCT4 was published in a preprint (63). They showed that E7, by downregulating MBD2 and upregulating TET1, induces hydroxymethylation and thus reactivation of OCT4.

### 1.4 Hepatitis B virus and pioneer factors

Hepatitis B virus (HBV) is a partially double-stranded DNA-virus of the hepadnaviridae family. The target cells of HBV are hepatocytes. After infection, the relaxed circular DNA (rcDNA) is repaired and transformed into stable covalently closed DNA (cccDNA). HBV is a major cause of hepatocellular carcinoma (HCC). Many different mechanisms have been reported by which HBV contributes to tumour progression, including viral integration into host DNA, genomic instability and modulation of various cancer-related pathways.

One pioneer factor that has been described to be upregulated in HBV+ HCC is the stem cell pluripotency factor OCT4 (57). Different mechanisms have been described how OCT4 upregulation contributes to HCC tumourigenesis. Li et al. reported that HBV and hepatitis C virus promote liver fibrosis through induction of TGF-β1. This TGF-β1 production activates pioneer factor OCT4 and NANOG. These two factors then activate pro-fibrotic genes such as α-SMA, TIMP-1 and Col1A1 and with this contributes to liver fibrosis (58). In addition, OCT4 was shown to mediate, together with STAT3, the re-expression of Sal-like protein 4 (SALL4), the expression of which is associated with poor prognosis in HCC (59). This upregulation of SALL4 is suggested to contribute to T-cell exhaustion. Mechanistically, SALL4 inhibits the transcription of the miRNA 200c, which, by upregulation of PD-L1, interferes in a crucial regulatory pathway of T-cell exhaustion (60).

Another factor that is upregulated in HCC, as in several other cancers, is Pax8. However, in HBV-associated HCC, the increased Pax8 levels have been shown to be directly modulated by the virus. Mechanistically, Pax8 is stabilized by the HBV X protein (HBx). This prevents its degradation, and the upregulated Pax8 levels have been shown to contribute to tumour progression in HCC (61).

### 1.5 Cell surface receptor regulation by pioneer factors

For a variety of human cancers, the expression of DUX4 in malignant cells has been reported. It has been demonstrated that DUX4 expression leads to a downregulation of MHC class I (MHC-I) expression from the cell surface. Chew et al. showed that DUX4 expression in solid cancers results in reduced expression of MHC-I in response to IFN-gamma (IFNγ) treatment (64). DUX4 protein interacts with STAT1 and broadly suppresses expression of IFNγ stimulated genes by decreasing bound STAT1 and Pol-II recruitment (65). Strikingly, DUX4 expression is mostly a feature of metastatic tumors and correlates with a bad tumor prognosis, reducing survival rates about a year compared to DUX4-negative tumors (66). The reason for that difference in tumor prognosis is most likely that DUX4 expression and MHC-I downregulation renders tumors less susceptible to treatment with checkpoint inhibitors. Similar observations have been made for HBV. Upon HBV infection, OCT4 induces SALL4 which results in upregulation of PD-L1. Patients with higher PD-L1 levels had worse prognosis and reduced overall survival, showing that this is critical for tumor immune evasion *in vivo* (67, 68). Interestingly, the pioneer factor PU.1 has also been implicated in the downregulation of MHCII after EBV infection (30). In EBV infected cells, LMP2 leads to downregulation of PU.1. PU.1 is a transcriptional activator of CIITA, and downregulation of PU.1 results in less CIITA expression and subsequently also MHC-II and CD47 downregulation. Whether this downregulation of MHC-I/MHC-II also plays a role during viral infection is currently unknown. One has to consider that all herpesviruses encode potent inhibitors that interfere with different aspects of antigen presentation via MHC-I/MHC-II. However, it is well conceivable that expression of pioneer factors contribute to herpesviral immune evasion in situations where viral MHC inhibitors are not able to execute their function, but this warrants further investigation.

## 2 Discussion

Viruses like Herpesviruses, Papillomaviruses and Hepatitis B viruses establish persistent infections in infected individuals. After primary infection and replication, the virus hides in niches with limited viral gene expression to prevent recognition by the host immune system and elimination of the infected cells. Herpesviruses for example establish life-long latency in long-lived cells of the blood or neuronal system. During latency the genome is silenced and subjected to epigenetic mechanism that influence gene expression from the viral genome. These include DNA-methylation and repressive histone modifications that prevent expression of genes that trigger the lytic replication cycle like BZLF1/BRLF1 for EBV and RTA/ZTA for KSHV. This allows the viruses to hide and persist in latent cells over years. However, occasionally the viruses have to initiate lytic replication. Lytic replication is triggered by a number of external stimuli that initiate the lytic cascade which results in production of progeny viruses. It seems that persistent viruses exploit the unique feature of pioneer factors to reverse the fate of epigenetically silenced viral chromatin in order to facilitate lytic replication. This has several advantages for the virus. First, it relies on established mechanisms of cellular proteins that are evolutionary conserved. Second, pioneer factors play very important roles in development and cell fate decisions. Therefore, there is limited room for mutations that might prevent exploitation by viruses. Any mutation that interferes with pioneer transcription factor function in the context of viral infection would also interfere with its original function and therefore be not compatible with development. Third, instead of regulating multiple cellular enzymes that revert silenced chromatin, the virus just has to ensure the expression of one cellular factor that does the job. The induction of a pioneer factor is therefore a simple way for the virus to induce transcription of epigenetically silenced viral chromatin. Particularly for persistent viruses like Herpesviruses, Papillomaviruses and also Hepatitis B viruses this is critical to exit the persistent state and re-enter the lytic replication cycle.

Some pioneer factors seem to be involved in transcriptional regulation of multiple viruses. DUX4 for example is not expressed in healthy adult tissue, but highly upregulated upon infection with human herpesviruses. In addition, we could show upregulation of DUX4 in single-cell RNA sequencing datasets of HPV-positive heck and neck cancer, indicating that DUX4 might also play a role in HPV infection. In addition, KLF4 has been shown to be important for activating transcription of EBV, HSV-1 and papillomaviruses. In particular for EBV and HSV-1, KLF4 seems to have similar roles. KLF4 activates expression of ICP4/ICP0 for HSV-1 and BRLF1/BZLF1 for EBV. Although the proteins are not homologous, they have similar functions in activating the lytic replication cascade. It would be very interesting to see whether DUX4 and KLF4 also play a role in the replication of other DNA-viruses or if other DNA-viruses use different pioneer factors as shown for HCMV which can be reactivated by FoxO1. For papillomaviruses, KLF4 expression is stabilized by posttranscriptional modification and binds to the genome. However, whether this activation directly contributes to HPV-induced cancer or is just a consequence of cell transformation is not known. As discussed above, DUX4 is also known upregulated in a variety of human cancers, and more work is needed to dissect the roles in virus induced cancer regarding viral replication and cancer development. In this context, it is also necessary to discuss whether pioneer factors act as pioneer factors under all circumstances or whether pioneer factors can also act as “normal” transcription factors. There is an ongoing debate regarding the exact differences between pioneer factors and conventional transcription factors. Hansen et al. used K562 lymphoblast cells and transfected them with FOXA1, a pioneer factor or HNF4A, a conventional transcription factor (69). Interestingly, only subtle differences in gene expression could be observed, indicating, that other factors like the number of transcription factor binding motifs contributes to the ability of a transcription factor to initiate transcription from silenced chromatin (69). Another interesting aspect is whether pioneer factor binding differs between cellular and viral chromatin. For example, during lytic viral replication, the viral genome is not associated with histones and therefore it might not be associated with pioneer factors that only bind to nucleosomal DNA. However, this might change over the course of viral infection and additional studies are needed to unravel the dynamic interplay between transcription factors and viral/cellular chromatin. In addition, it would be worthwhile to investigate if some viral transactivator proteins also fulfill the criteria of pioneer factors. By definition, pioneer factors i) bind nucleosomal DNA *in vivo* and *in vitro* ii) induce chromatin remodeling around DNA binding sites and iii) induce global epigenetic changes in the cell, but this has not been investigated in detail. Interestingly, the HSV-1 transactivator ICP4 preferentially binds to chromatin that is not associated with nucleosomes to discriminate between viral and cellular chromatin (70).

Overall, there is growing evidence that pioneer factors play important roles in regulating transcription from the viral genome. Especially DNA-viruses that have to switch between a dormant, persistent state and lytic replication rely on pioneer factors like KLF4, DUX4 and probably others to revert the fate of epigenetically silenced viral chromatin.

## Author contributions

EN: Writing – original draft, Writing – review & editing, Conceptualization. AB: Visualization, Writing – review & editing. DW: Writing – review & editing, Conceptualization. FF: Writing – original draft, Writing – review & editing, Conceptualization.
